# Rosuvastatin Treatment Affects Both Basal and Glucose-Induced Insulin Secretion in INS-1 832/13 Cells

**DOI:** 10.1371/journal.pone.0151592

**Published:** 2016-03-17

**Authors:** Vishal A. Salunkhe, Olof Elvstam, Lena Eliasson, Anna Wendt

**Affiliations:** Unit of Islet Cell Exocytosis, Lund University Diabetes Centre, Dept. Clinical Sciences in Malmö, Lund University, Clinical Research Centre, SUS Malmö, Malmö, Sweden; Institut d'Investigacions Biomèdiques August Pi i Sunyer, SPAIN

## Abstract

Rosuvastatin is a member of the statin family. Like the other statins it is prescribed to lower cholesterol levels and thereby reduce the risk of cardiovascular events. Rosuvastatin lowers the cholesterol levels by inhibiting the key enzyme 3-hydroxy-3-methyl-glutaryl-CoA reductase (HMG-CoA reductase) in the cholesterol producing mevalonate pathway. It has been recognized that apart from their beneficial lipid lowering effects, statins also exhibit diabetogenic properties. The molecular mechanisms behind these remain unresolved. To investigate the effects of rosuvastatin on insulin secretion, we treated INS-1 832/13 cells with varying doses (20 nM to 20 μM) of rosuvastatin for 48 h. At concentrations of 2 μM and above basal insulin secretion was significantly increased. Using diazoxide we could determine that rosuvastatin did not increase basal insulin secretion by corrupting the K_ATP_ channels. Glucose-induced insulin secretion on the other hand seemed to be affected differently at different rosuvastatin concentrations. Rosuvastatin treatment (20 μM) for 24–48 h inhibited voltage-gated Ca^2+^ channels, which lead to reduced depolarization-induced exocytosis of insulin-containing granules. At lower concentrations of rosuvastatin (≤ 2 μM) the stimulus-secretion coupling pathway was intact downstream of the K_ATP_ channels as assessed by the patch clamp technique. However, a reduction in glucose-induced insulin secretion could be observed with rosuvastatin concentrations as low as 200 nM. The inhibitory effects of rosuvastatin on glucose-induced insulin secretion could be reversed with mevalonate, but not squalene, indicating that rosuvastatin affects insulin secretion through its effects on the mevalonate pathway, but not through the reduction of cholesterol biosynthesis. Taken together, these data suggest that rosuvastatin has the potential to increase basal insulin secretion and reduce glucose-induced insulin secretion. The latter is possibly an unavoidable side effect of rosuvastatin treatment as it occurs through the same mechanisms as the lipid-lowering effects of the drug.

## Introduction

Rosuvastatin, like all other statins, is a cholesterol lowering drug prescribed to reduce the risk of cardiovascular disease. Rosuvastatin acts by inhibiting 3-hydroxy-3-methyl-glutaryl-CoA reductase (HMG-CoA reductase), which is the rate-limiting enzyme in the biosynthesis of cholesterol through the mevalonate pathway. In general statins are considered safe and their ability to reduce cardiovascular events is well documented [1]. However, in the “Justification for the Use of Statins in Prevention: an Intervention Trial Evaluating Rosuvastatin” (JUPITER trial) physician-reported diabetes among patients given rosuvastatin was significantly increased compared to placebo control [2]. Other studies have also documented the diabetogenic effects of statins highlighting rosuvastatin as one of the more diabetogenic ones [3].

Due to its particular chemical structure rosuvastatin is one of the most potent inhibitors of HMG-CoA reductase. It is hydrophilic in nature and is actively transported into the hepatocytes through membrane bound transporters e.g. Organic Anion Transporting Polypeptides (OATP) [4]. It is believed that rosuvastatin enters non-hepatic tissue only to a low extent [5]. However, functional OATP1B3 has recently been found in pancreatic beta cells [6] providing a pathway through which rosuvastatin can enter these cells. The underlying mechanisms behind why statins cause diabetes are unclear but adverse effects on both insulin secretion and insulin resistance have been suggested [7].

Insulin is the major glucose-lowering hormone in the body and as such its release is tightly regulated. The main trigger for insulin release is an increase in blood glucose. Blood glucose equilibrates across the beta cell membrane via low-affinity glucose transporters. Inside the beta cell, glucose is metabolized and the resulting increase in the ATP/ADP ratio closes ATP-sensitive K^+^ channels (K_ATP_ channels). Closure of the K_ATP_ channels initiate a depolarization of the cell membrane which ultimately leads to the opening of voltage-gated Ca^2+^ channels. The resulting influx of Ca^2+^ triggers exocytosis of insulin containing granules and thereby insulin is released. The chain of events outlined above is collectively referred to as the stimulus-secretion coupling of the beta cells [8]. Insulin secretion can be augmented by several mechanisms including the presence of incretins such as GLP-1 and GIP [9] as well as potentiation of insulin secretion via the amplifying pathway of glucose [8].

The effects of statins on insulin secretion and the stimulus-secretion coupling of the beta cells are uncertain, but in the Metabolic Syndrome in Men (METSIM) cohort [10, 11] statins, including rosuvastatin, reduce insulin secretion. On a molecular level simvastatin has been reported to reduce the Ca^2+^ current through L-type voltage-gated Ca^2+^ channels in primary rat beta cells [12] and lovastatin has been found to impair bombesin- and vasopressin-induced amplification of insulin secretion, probably through small GTP-binding proteins [13]. It has also been hypothesized that statins reduce the production of ATP [14].

Here we have investigated the effects of short-term (24-48h) incubations with rosuvastatin on insulin release and the stimulus-secretion coupling of the beta cells.

## Material and Methods

### Materials

All chemicals were purchased from Sigma Aldrich(MO, USA) unless otherwise indicated.

### Cell culture

**Fig 1:**
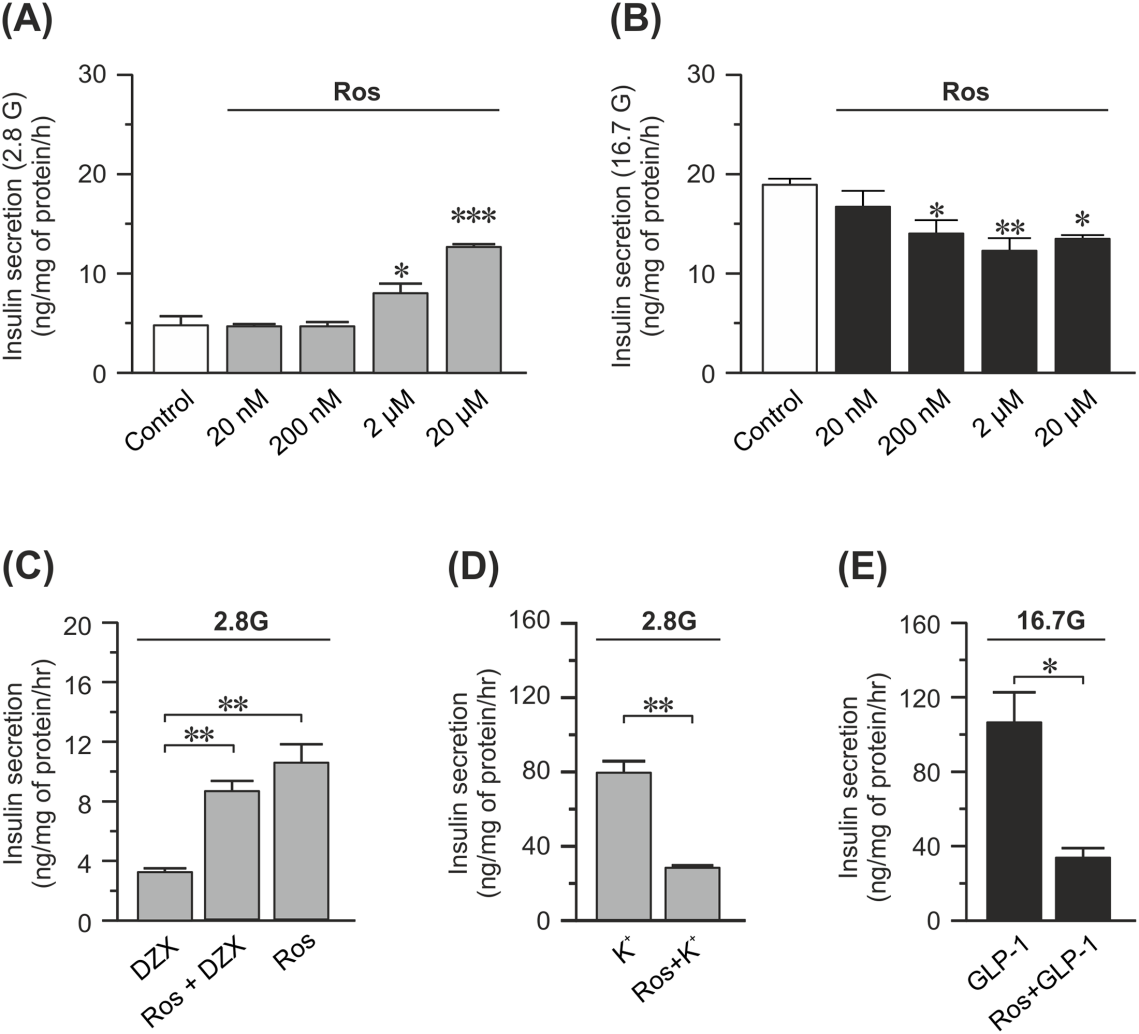
Effects of 48 h of rosuvastatin treatment on insulin secretion in INS-1 832/13 cells. (A) Insulin secretion at 2.8 mM glucose measured in the presence of rosuvastatin (Ros) at concentrations ranging from 20 nM-20 μM as indicated in the figure. Statistical significance is calculated compared to the control (DMSO). (B) Same as in (A) but insulin secretion is measured at 16.7 mM glucose instead. (C) Insulin secretion at 2.8 mM glucose from cells treated with 20μM rosuvastatin, 200 μM diazoxide (Dzx) or a combination of the two. (D) Insulin secretion at 2.8 mM glucose and 50 mM K^+^ with and without 20μM rosuvastatin. (E) Insulin secretion at 16.7 mM glucose from cells treated with 100 nM GLP-1 with and without 20 μM rosuvastatin. Data are given as mean ± SEM from 3 experiments with 3 technical replicates in each experiment. * p≤ 0.05; ** p≤ 0.01; *** p≤ 0.001.

**Fig 2:**
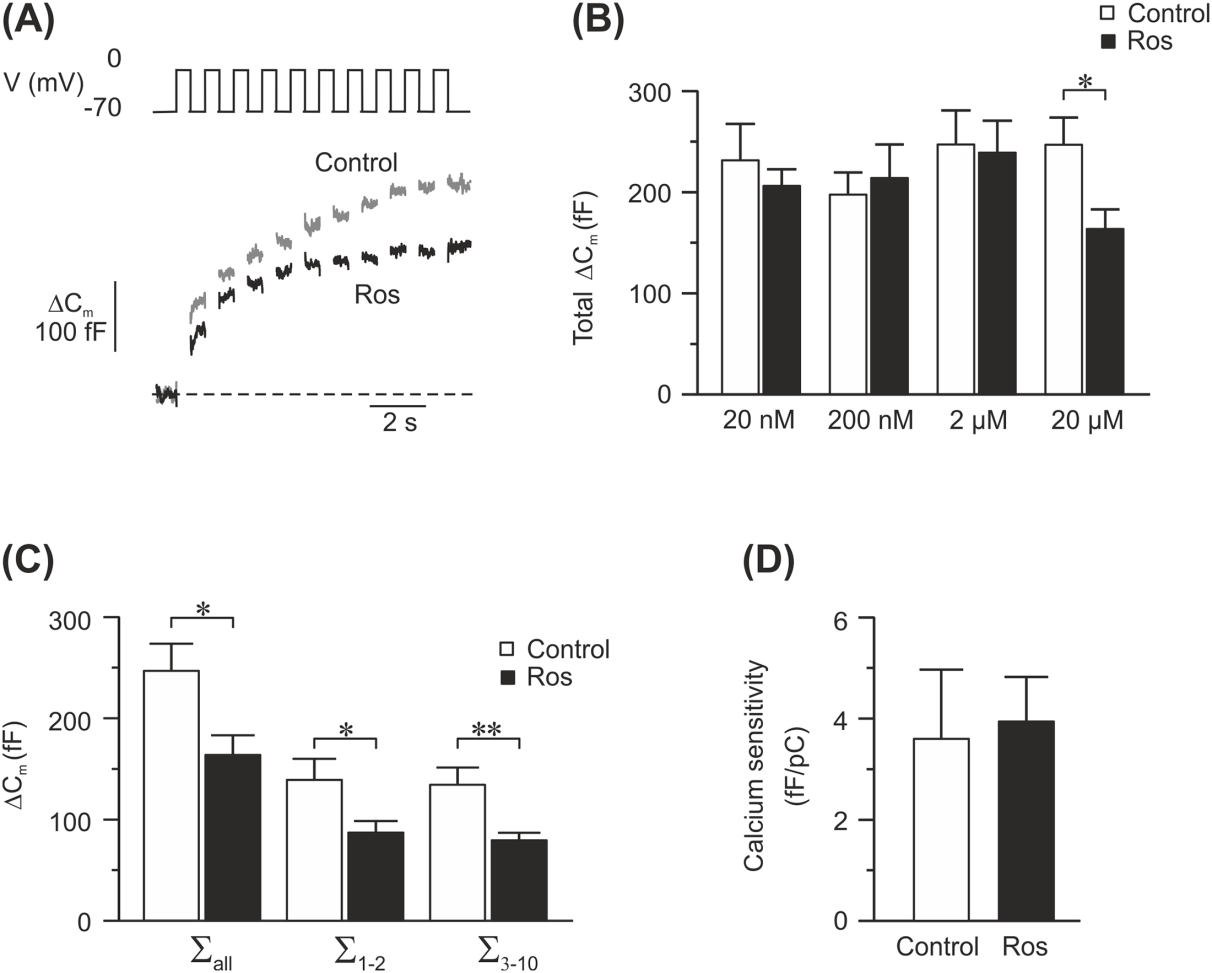
Effects of 24–48 h of rosuvastatin treatment on exocytosis in INS-1 832/13 cells. (A) Example traces of depolarization-induced exocytosis measured as changes in cell membrane capacitance, from rosuvastatin-treated cells (20 μM; black trace) and control cells (grey trace). Exocytosis was evoked by a train of ten 500 ms depolarizing pulses from -70 mV to 0 mV. (B) Summary of the total capacitance change during the train in control cells (white bars) and rosuvastatin-treated cells (Ros; black bars). The concentration of rosuvastatin in these experiments ranged from 20 nM-20 μM as marked in the figure. (C) A graph describing the exocytotic response to all 10 pulses (∑_all_) to the first 2 pulses (∑_1–2_) or to the latter 8 pulses (∑_3–10_) in cells incubated with 20 μM rosuvastatin (black bars) and their controls (white bars). (D) Calcium sensitivity of cells incubated with 20 μM rosuvastatin (Ros; black bars) or their controls (white bars). Calcium sensitivity is calculated by dividing the exocytotic response to the first pulse with the calcium charge measured during the same pulse. Data are given as mean ± SEM of 21–31 cells. * p≤ 0.05; ** p≤ 0.01.

**Fig 3:**
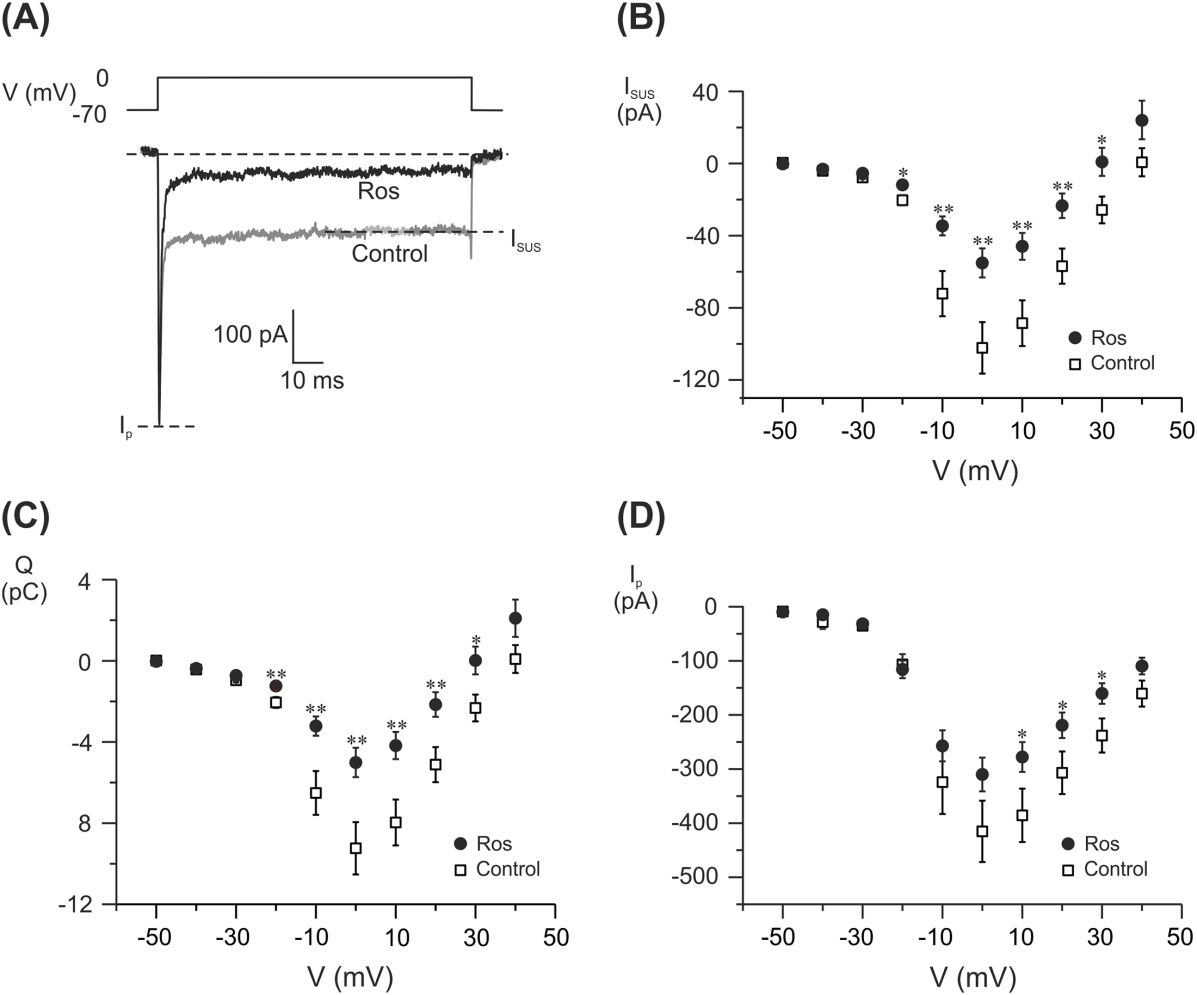
Electrophysiological characterization of voltage-gated ion channels in rosuvastatin treated INS-1 832/13. Cells were treated with 20 μM rosuvastatin for 24-48h. (A) Example traces of currents evoked by a depolarization to 0 mV in a single rosuvastatin-treated (Ros; black trace) and control (grey trace) cell. I_sus_ and I_p_ measured in (B) and (D) are marked. (B) Sustained current (I_sus_)-voltage (V) relationship (C) charge (Q)-voltage (V) relationship. Charge is measured as the area enclosed by the curve in (A). (D) peak current (I_p_)-voltage (V) relationship in INS-1 832/13 cells treated with 20 μM rosuvastatin (Ros; black dots) or control cells (Control; white squares). Data are given as mean ± SEM of 28–38 cells.

**Fig 4:**
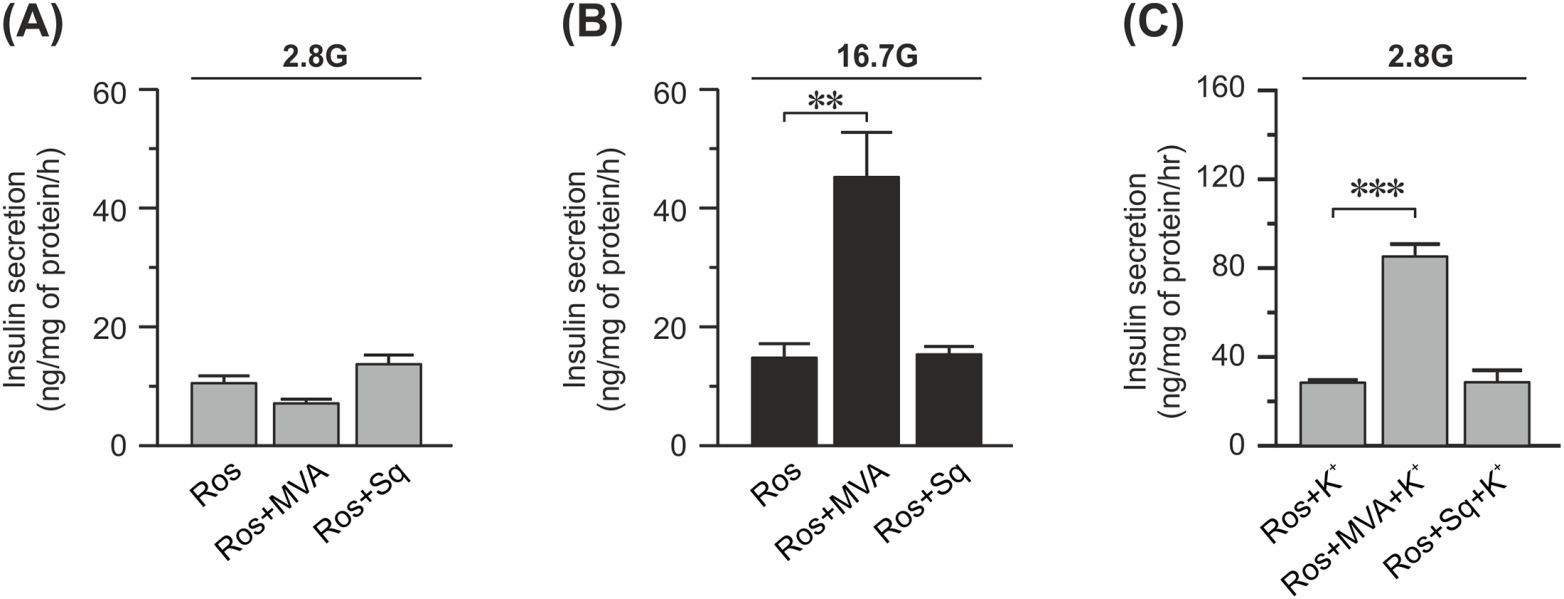
Effect of mevalonate and squalene on insulin secretion from rosuvastatin-treated INS-1 832/13 cells. (A) Insulin secretion at basal (2.8 mM) concentrations of glucose from cells treated with 20 μM rosuvastatin (Ros), 50 μM mevalonate (MVA), and 100 μM squalene (Sq) for 48 h as indicated in the figure. (B) Same as in (A) but with stimulatory (16.7 mM) concentrations of glucose instead. (C) Same as in (A) but the cells are stimulated with 50 mM K^+^ as well. Data are given as mean ± SEM from 3 experiments with 3 technical replicates in each experiment. * p≤ 0.05.

The pancreatic β-cell line INS-1 832/13 [15] was maintained in RPMI 1640 medium containing 11.1 mM D-glucose (HyClone, UT, USA) and supplemented with 10% (v/v) heat inactivated fetal bovine serum, 100 IU/ml of penicillin (HyClone, UT, USA), 100 μg/ml of streptomycin (HyClone, UT, USA), 10 mM HEPES (HyClone, UT, USA), 2 mM L-glutamine (HyClone, UT, USA), 1 mM sodium pyruvate (HyClone, UT, USA) and 50 μM 2-mercaptoethanol Sigma Aldrich (MO, USA). The cells were routinely reseeded every 2–3 days and kept in a humidified cell incubator at 37°C with 5% CO_2_. Rosuvastatin (LKT Laboratories, MN, USA) was dissolved in dimethyl sulfoxide (DMSO). Final concentrations of rosuvastatin ranged from 20 nM to 20 μM as indicated in the Figs 1, 2, 3 and 4. INS-1 832/13 cells were incubated with rosuvastatin, mevalonate and/or squalene for 48 h prior to insulin secretion experiments and in rosuvastatin 24–48 h prior to electrophysiological measurements.

### Glucose-stimulated insulin secretion

These experiments were essentially performed as previously described [16]. For each condition INS-1 832/13 cells were seeded in triplicates in a 24-well plate. At a confluence of ~100% the cells were gently washed with freshly made Secretion Assay Buffer (SAB) pH 7.2, supplemented with 2.8 mM glucose (for the preparation of SAB see below). The cells were pre-incubated in fresh SAB supplemented with 2.8 mM glucose for 2h followed by 1h incubation in SAB supplemented with 2.8, 8.3 or 16.7 mM glucose with or without 50 mM K^+^, 200 μM diazoxide, 100 nM GLP-1, 50 μM mevalonate, or 100 μM squalene as indicated in the figures. All incubations were performed at 37°C in a humidified cell incubator with 5% CO_2_. At the end of the experiment an aliquot of the supernatant was carefully collected from each well. Insulin levels were measured according to the manufacturer using Coat-a-Count RIA (Millipore Corporation, MA, USA; Fig 1A and 1B) or Mercodia Insulin ELISA (Mercodia, Uppsala, Sweden; Figs 1C–1E and 4). SAB consists of (in mM): 114 NaCl, 4.7 KCl, 1.2 KH_2_PO_4,_ 1.16 MgSO_4_, 20 HEPES, 25.5 NaHCO_3_, 2.5 CaCl_2_ and 0.2% BSA. When 50 mM K^+^ was used Na^+^ was equally reduced to keep the osmolarity. Insulin secretion data were normalized to total protein content in the same well.

For protein content, the proteins were extracted by washing the cells in ice cold PBS after which they were incubated on ice for 15 minutes in cold RIPA buffer (150 mM NaCl, 1% TritonX-100, 0.1% SDS, 50 mM Tris-Cl, pH 8) supplemented with EDTA-free protease inhibitor (Roche, NJ, USA). The lysed cells were loosened by pipetting and the resulting lysate was transferred to pre-cooled tubes and centrifuged for 15 minutes at 4°C; 14 000 x g. The supernatant was then collected and stored at -20°C. Protein content was determined with a BCA assay (Pierce, IL, USA) which was analyzed on a BioRad Model 6870 Microplate Reader. For viewing purposes we split two sets of experiments into several graphs. Fig 1A and 1B where performed at the same time. Figs 1C–1E and 4 belongs to a second set of experiments and here the same data is used in more than one graph.

### Electrophysiology

Patch pipettes were pulled from borosilicate glass capillaries, coated with sticky wax (Kemdent, UK) and then fire-polished. The pipette resistance was ~3–6 MΩ when the pipettes were filled with the intracellular solutions specified below. The standard whole-cell configuration of the patch clamp technique was used in all experiments. The protocol was as follows; ≥ 1 min after establishment of the whole-cell configuration a train of ten depolarizations from -70 mV to 0 mV was applied. The cell was then allowed to recover for ≥ 30 s where after Ca^2+^ currents were evoked by depolarizations from a resting potential of -70 mV to voltages ranging between -50 mV to +40 mV. Changes in membrane capacitance and whole-cell currents were recorded using an EPC-10 patch-clamp amplifier and the software Patchmaster (Heka Elektronik, Germany; version 2–73). Changes in cell capacitance during the train of ten depolarizations were measured using the software-based lock-in application that adds sinewaves with a frequency of 500 Hz to the holding potential of the amplifier. The standard extracellular solution consisted of (in mM): 118 NaCl, 20 tetraethyl-ammonium chloride (TEA-Cl; to block K^+^ currents), 5.6 KCl, 2.6 CaCl_2_, 1.2 MgCl_2_, 5 glucose and 5 HEPES (pH 7.4 using NaOH). The pipette solutions consisted of (in mM) 125 Cs-Glut, 10 NaCl, 10 CsCl, 1 MgCl_2_, 0.05 EGTA, 3 Mg-ATP, 5 HEPES (pH 7.15 using CsOH) and 0.1 cAMP. The control cells in Fig 2 were treated with DMSO at a concentration matching that of the rosuvastatin-treated cells. To exclude the Na^+^ current in Fig 3C, the area is measured 2 ms after the onset of the pulse until the end of the pulse.

### Data analysis

Data are represented as means ± SEM. Statistical significance was determined using one-way ANOVA with a Dunnet test to correct for multiple comparisons in Figs 1A, 1B and 4A–4C. In Fig 1C one-way ANOVA with a Tukey test to correct for multiple comparisons was used. In all other figures statistical significance was determined with two-tailed Student’s *t*-test.

## Results

### Rosuvastatin decrease glucose-induced insulin secretion and increase basal insulin secretion

First we investigated the dose-response curve of rosuvastatin on basal and glucose-induced insulin secretion. We found that in INS-1 832/13 cells 20 nM rosuvastatin had no effect on either basal (2.8 mM glucose; Fig 1A) or glucose-induced (16.7 mM glucose; Fig 1B) insulin secretion. Incubation with ≥ 200 nM rosuvastatin reduced glucose-induced insulin secretion by ~25%. Interestingly, 2μM rosuvastatin markedly increased basal insulin secretion by ~65% and after incubation in 20 μM rosuvastatin basal and glucose-induced insulin secretion approached each other (Fig 1A and 1B).

Next we wanted to examine the background to the increased basal insulin secretion with 20 μM rosuvastatin. The molecular mechanisms behind basal insulin secretion are unknown. Here, we decided to investigate if the K_ATP_ channel opener diazoxide could modulate the effects of rosuvastatin on basal insulin secretion. The K_ATP_ channel has a major influence on the membrane potential of the β-cell and we reasoned that premature closure of this channel could initiate cell membrane depolarization and insulin secretion. Our data show that the increased basal insulin secretion induced by rosuvastatin was not significantly reduced in the presence of diazoxide (Fig 1C). This suggests that rosuvastatin does not act on the properties of the K_ATP_ channel.

Thereafter we investigated the inhibitory effect of rosuvastatin on glucose-induced insulin secretion. To determine if rosuvastatin acts downstream of the K_ATP_ channel in the stimulus-secretion coupling pathway we performed experiments with high (50 mM) concentration of extracellular K^+^ in the presence of 2.8 mM glucose. Rosuvastatin treatment significantly reduced insulin secretion also under these conditions (Fig 1D).

Last we investigated if potentiation of glucose-induced insulin secretion with the incretin GLP-1 could restore insulin secretion in rosuvastatin-treated cells. We found that rosuvastatin significantly reduced insulin secretion also in the presence of GLP-1 (Fig 1E).

### High doses of rosuvastatin decrease depolarization induced exocytosis

To determine where in the stimulus-secretion coupling pathway of the beta cell rosuvastatin acts, we investigated exocytosis in rosuvastatin-treated cells. We did so by performing patch clamp experiments on INS-1 832/13 cells pre-incubated for 24–48 h with different doses of rosuvastatin. Exocytosis was elicited by Ca^2+^-influx generated by a train of ten depolarizing pulses from -70 mV to 0 mV and measured as changes in cell capacitance (Fig 2A). At 20 μM rosuvastatin the total increase in cell capacitance elicited by the train was reduced by 34% compared to control (164 ± 19 fF *vs* 247 ± 27 fF; n = 27–28; P < 0.05; Fig 2B). The reduction in the exocytotic response was equal across the depolarizing pulses in the train (Fig 2C). No other rosuvastatin concentration tested changed the exocytotic response to the applied train (Fig 2B). By comparing the exocytotic response evoked by the first depolarizing pulse in the train with the Ca^2+^ current elicited by the same pulse, we can determine the Ca^2+^ sensitivity of the exocytotic response. As can be seen in Fig 2D there is no difference in Ca^2+^ sensitivity between the control cells and those treated with 20 μM rosuvastatin. Similarly, we did not detect a change in Ca^2+^ sensitivity at any other rosuvastatin concentration tested (*data not shown*). Exocytosis of insulin granules is a Ca^2+^ dependent process [17]. The estimation of Ca^2+^ sensitivity tells us how well the exocytotic machinery can sense and respond to the voltage-induced increase in intracellular Ca^2+^ concentrations. The similarities in Ca^2+^ sensitivity between the two groups indicates that the reduced exocytotic response in rosuvastatin (20 μM) treated cells does not lie downstream of the voltage-gated Ca^2+^ channels.

### High doses of rosuvastatin decrease Ca^2+^ currents through voltage-gated Ca^2+^ channels

The calcium sensitivity data indicated that rosuvastatin changes the influx of Ca^2+^ ions through voltage-gated Ca^2+^ channels. We therefore performed an electrophysiological investigation of these channels after 24–48 h of rosuvastatin treatment. From a holding potential of -70 mV currents were evoked by membrane depolarizations from -50 mV to +40 mV. We then analyzed the obtained data for peak current (Ip), sustained current (Isus) and charge (Q) as outlined in Fig 3A. In these experiments Ip mainly reflects the Na^+^ current while Isus and Q reflect the Ca^2+^ current. We found no difference in Ip at any conditions tested. However, we saw a ~45% reduction in Isus at 0 mV with 20 μM rosuvastatin. This was accompanied by a ~45% reduction in Q (Fig 3B and 3C). These data clearly shows that we have a reduced influx of Ca^2+^ through voltage-gated Ca^2+^ channels while the influx of Na^+^ through the voltage-gated Na^+^ channels remains unaffected (Fig 3D).

### Mevalonate but not squalene rescues the secretory defects caused by rosuvastatin

Statins acts by inhibiting the production of mevalonate from HMG-CoA. The formation of mevalonate is upstream in a series of reactions, collectively referred to as the mevalonate pathway, that ultimately leads to the formation of cholesterol as well as other compounds such as sterols, ubiquinones and prenylated proteins [18]. Squalene is found further downstream in this pathway in one of the arms that exclusively leads to the formation of cholesterol. We added mevalonate or squalene to cells treated with 20 μM rosuvastatin for 48 h in order to determine if rosuvastatin acts on insulin secretion via the cholesterol synthesis pathway.

Rosuvastatin-induced defects in insulin secretion at 2.8 mM glucose were not rescued by applying either mevalonate or squalene (Fig 4A). However, the rosuvastatin-induced defects in both glucose induced (16.7 mM glucose) and depolarization-induced (2.8 mM glucose and 50 mM K^+^) secretion were rescued by mevalonate, but not by squalene (Fig 4B and 4C). This indicates that glucose-induced but not basal insulin secretion is compromised by rosuvastatin through the direct effects of the drug on the non-cholesterol forming parts of the mevalonate pathway.

## Discussion

Statins are highly prescribed to prevent cardiovascular disease, the leading cause of death in men and women worldwide. Although clearly lifesaving, statins are not without side effects. In recent years it has been recognized that statins increase the risk of type 2 diabetes [3]. In this study we have focused on the statin rosuvastatin since the literature indicates rosuvastatin as one of the more diabetogenic statins [3]. We found that rosuvastatin treatment increase basal insulin secretion and decrease glucose-stimulated insulin secretion. The etiology of the increased basal insulin secretion is still uncertain while the decrease in glucose-stimulated insulin secretion is a byproduct of the inhibitory effects of rosuvastatin on the enzyme HMG-CoA reductase and the mevalonate pathway. Interestingly, it is not related to the cholesterol lowering effects of the drug.

Our study, conducted on the well-established pancreatic beta cell line INS-1 832/13 [15], shows that short time (48 h) incubation with 200 nM rosuvastatin results in a reduced glucose-stimulated insulin secretion (Fig 1B). This is in contrast to what has been reported by Ishikawa *et*. *al*. These authors performed a study similar to ours, but on the statins pravastatin, atorvastatin and simvastatin and in MIN-6 cells [19]. In the latter study, there was no decreased glucose-induced insulin secretion at any concentration tested with either of the statins. This might indicate that there is a difference between the statins with respect to how they affect beta cell function, although we cannot rule out that the different choice of cell line also plays a role.

In the present study, treatment with higher concentrations (≥ 2 μM) of rosuvastatin results in an increased basal insulin secretion (Fig 1A). Similar effects of other statins have been shown before [19]. It is unknown which mechanisms that underlie basal insulin secretion, but we can show here that opening the K_ATP_ channels using diazoxide did not rescue the phenotype (Fig 1C). Hence, the increased basal insulin secretion is not due to premature closure of the K_ATP_ channels. Nor does it appear to be a direct side effect of inhibiting the mevalonate pathway since addition of mevalonate or squalene fails to normalize basal insulin secretion in these cells (Fig 4). Therefore, it appears that the increase in basal insulin secretion is due to, as of yet, unidentified off target effects of the drug. It is interesting that statins increase basal insulin secretion, since increased basal insulin secretion occurs in type 2 diabetic patients [20]. It has also been suggested that increased basal insulin secretion leads to increased food consumption, obesity and diabetes [21].

Admittedly, all effects on insulin secretion seems to occur at fairly high statin concentrations compared to the average plasma concentration of ~20 nM which has been reported for individuals on the highest therapeutic dose, 40 mg, of rosuvastatin (calculated from the values reported by DeGorter *et*. *al*. [22]). However, it is not unconceivable that long-term exposure to lower levels of statins might have the same effect; this is an area that should be further explored in other systems. In this context it is noteworthy that a substantial variability (45-fold) in the plasma levels of rosuvastatin between individuals on the same dose has been reported [22]. This variability was attributed to genetic polymorphism in relevant transporter proteins. Also, there are studies showing that individuals from Asia experience ~2 fold higher systemic concentrations of rosuvastatin after administration compared to individuals born and resident in Western countries [23]. Taken together this opens up for substantial inter-individual differences regarding the risks of adverse effects from statin treatment.

Glucose-induced insulin secretion is depolarization induced [24]. In this study we mimicked this by depolarizing the cell membrane both directly with the patch clamp technique and via high extracellular concentrations of K^+^. Even strong depolarizations to 0 mV (Fig 2B) could not cancel out the inhibitory effects of rosuvastatin (20 μM) on insulin granule exocytosis. Remember that in these experiments the K_ATP_ channels are bypassed by the depolarization. However, 200 nM rosuvastatin reduces glucose-stimulated insulin secretion without affecting depolarization induced exocytosis (Figs 1B and 2B). Taken together this indicates that processes both upstream (including amplifying pathways) and downstream of the K_ATP_ channels are affected by rosuvastatin depending on the concentration used. Patch clamp experiments showed reduced Ca^2+^ currents through the voltage-gated Ca^2+^ channels at 20 μM of rosuvastatin, similarly to what has previously been reported with simvastatin [12]. The reduction in Ca^2+^ current was mirrored by a decrease in exocytosis of insulin-containing granules with a maintained Ca^2+^ sensitivity (Fig 2). Higher concentrations of rosuvastatin are required for an effect on the voltage-gated Ca^2+^ channels and exocytosis compared to what is needed for an effect on glucose-induced insulin secretion (Fig 1B). The reason for this is not clear. In patch clamp experiments all steps in the stimulus-secretion coupling pathway prior to the opening of voltage-gated Ca^2+^ channels are bypassed by the depolarization. In addition the pipette solution, which is infused into the cell in the standard whole-cell configuration used here, contains ATP. Indeed, rosuvastatin has been shown to decrease the plasma levels of coenzyme Q10 [25]. Coenzyme Q10 serves several important functions in the cells including a role in mitochondrial ATP-production. It is therefore reasonable to believe that reduction of coenzyme Q10 will lead to a reduced ATP production in the beta-cell which in turn would affect the proper closure of the K_ATP_ channels. A reduction of ATP through the reduction of coenzyme Q10 levels cannot be detected with the patch clamp method since we have ATP present in the pipette solution and the closure of the K_ATP_ channels is bypassed by the depolarization. However, when measuring glucose-induced insulin secretion, manipulation of ATP levels would affect insulin secretion. We cannot provide evidence that rosuvastatin lowers glucose-induced insulin secretion through altered ATP production as has been suggested [26] but it is not unlikely that this, at least in part, explains why glucose-induced insulin secretion is reduced at rosuvastatin concentrations below those that affect voltage-gated Ca^2+^ channels and exocytosis.

Yada *et al*. reports immediate effects of simvastatin on the voltage-gated Ca^2+^ channels [12], and Yaluri et al report that short (1–2 h) incubations with simvastatin reduce insulin secretion [27]. Both of these findings are conducted with simvastatin instead of rosuvastatin, but as all statins function by inhibiting HMG-CoA reductase they still function as an argument against that the effect goes via the mevalonate pathway. We have not conducted direct experiments to solve this issue, however we did find that glucose-induced insulin secretion from rosuvastatin-treated cells is rescued by mevalonate but not squalene. Note that in our experiments the cells are incubated for substantially longer time (48 h) with the statin which will allow for slower mechanisms to take place. Our findings are consistent with the hypothesis that the effect of rosuvastatin on voltage-gated Ca^2+^ channels, at least after 48 h of incubation, acts through the mevalonate pathway although not through the reduction of cholesterol. As a result, we believe that the reduction of glucose-induced insulin secretion is not due to off-target effects of the drug but rather side effects of its effective blockage of the mevalonate pathway.

One limitation of this study is that it is conducted in a clonal cell line. A direct extrapolation of the data retrieved here on i.e the human setting is not easily done due to the many differences between the systems. For instance it has been reported that short term (24 h) application of 100 nM rosuvastatin to human islets has no deleterious effects on insulin secretion [28] which is in direct contrast to what we report here. We also acknowledge the difficulties in mimicking long term *in vivo* applications of a drug by short term *in vitro* applications. Nevertheless, we believe that to deliver a proof of principle INS-1 832/13 cells serves as a useful model system. The fact that rosuvastatin affects insulin secretion in INS-1 832/13 cells makes it highly interesting to investigate this phenomenon further in human islets. At which concentrations of the drug this might occur in the human body and at what rate is difficult to predict by the experiments performed here. Since the risk to develop reduced insulin secretion and type 2 diabetes for humans on statin treatment is dose-dependent [11], it is interesting that we find different effects on the insulin secretory machinery at different doses of rosuvastatin. Our data indicates that rosuvastatin-induced adverse effects on glucose-induced insulin secretion are caused by the very blockage of the mevalonate pathway by the statin. We can only speculate about which products of the mevalonate pathway that are responsible for the adverse effects. Cofactor Q10, which has been mentioned before, as well as prenylated proteins are produced through the mevalonate pathway, but not through squalene. It was recently published that short (2h) incubations with simvastatin in INS-1E cells lowers glucose-induced insulin secretion through the reduction of prenylated proteins [29]. Prenylated proteins include G-coupled proteins such as the Rab proteins. Several of these proteins are involved in mobilization and priming of insulin vesicles. A defect in these steps would be measurable both in patch clamp experiments and in secretion experiments. In this context it is interesting that the Rab effector protein RIM (Rab-3 interacting molecule) [30] has been shown to connect to voltage-gated Ca^2+^ channels in secretory cells [31, 32]. Effector protein in this context refers to a protein that responds to a specific Rab and mediates at least some of its downstream effects [33]. RIM is also present in pancreatic beta cells including INS-1E cells where it plays a role in the regulation of insulin secretion [34]. It is therefore tempting to speculate that the reduction of glucose-induced insulin secretion in our experiments is partly due to the reduction of prenylated proteins such as Rab proteins.

We conclude that rosuvastatin, through its efficient inhibition of the mevalonate pathway, adds several defects to the secretory machinery through largely unidentified pathways. These defects in combination with other weaknesses already present in the insulin-signaling pathway in diabetic-proned patients might explain much of the reported increased risk of developing type 2 diabetes in rosuvastatin treated patients.
